# The Quality of Working Life Questionnaire for Cancer Survivors (QWLQ-CS): a Pre-test Study

**DOI:** 10.1186/s12913-016-1440-4

**Published:** 2016-06-02

**Authors:** Merel de Jong, Sietske J. Tamminga, Angela G.E.M. de Boer, Monique H.W. Frings-Dresen

**Affiliations:** Coronel Institute of Occupational Health, Academic Medical Center, University of Amsterdam, P.O. Box 22660, 1100 DD Amsterdam, The Netherlands

**Keywords:** Quality of working life, Cancer survivors, Questionnaire, Return-to-work, Work continuation

## Abstract

**Background:**

Returning to and continuing work is important to many cancer survivors, but also represents a challenge. We know little about subjective work outcomes and how cancer survivors perceive being returned to work. Therefore, we developed the Quality of Working Life Questionnaire for Cancer Survivors (QWLQ-CS). Our aim was to pre-test the items of the initial QWLQ-CS on acceptability and comprehensiveness. In addition, item retention was performed by pre-assessing the relevance scores and response distributions of the items in the QWLQ-CS.

**Methods:**

Semi-structured interviews were conducted after cancer survivors, who had returned to work, filled in the 102 items of the QWLQ-CS. To improve acceptability and comprehensiveness, the semi-structured interview inquired about items that were annoying, difficult, confusing, twofold or redundant. If cancer survivors had difficulty explaining their opinion or emotion about an item, the interviewer used verbal probing technique to investigate the cancer survivor’s underlying thoughts. The cancer survivors’ comments on the items were analysed, and items were revised accordingly. Decisions on item retention regarding the relevance of items and the response distributions were made by means of pre-set decision rules.

**Results:**

The 19 cancer survivors (53 % male) had a mean age of 51 ± 11 years old. They were diagnosed between 2009 and 2013 with lymphoma, leukaemia, prostate cancer, breast cancer, or colon cancer. Acceptability of the QWLQ-CS was good - none of the items were annoying - but 73 items were considered difficult, confusing, twofold or redundant. To improve acceptability, for instance, the authors replaced the phrase ‘disease’ with ‘health situation’ in several items. Consequently, comprehensiveness was improved by the authors rephrasing and adjusting items by adding clarifying words, such as ‘in the work situation’. The pre-assessment of the relevance scores resulted in a sufficient number of cancer survivors indicating the items as relevant to their quality of working life, and no evident indication for uneven response distributions. Therefore, all items were retained.

**Conclusions:**

The 104 items of the preliminary QWLQ-CS were found relevant, acceptable and comprehensible by cancer survivors who have returned to work. The QWLQ-CS is now suitable for larger sample sizes of cancer survivors, which is necessary to test the psychometric properties of this questionnaire.

**Electronic supplementary material:**

The online version of this article (doi:10.1186/s12913-016-1440-4) contains supplementary material, which is available to authorized users.

## Background

As the prevalence of cancer is rising, the number of cancer survivors participating in the labour market is increasing accordingly [1]. The growth in cancer prevalence is caused by early detection of cancer, improved treatment, and increasing age among cancer survivors [2]. The increasing retirement age is also causing more cancer survivors to face the challenges of having cancer while participating in the labour market [3].

Work provides control and a sense of normality, and employment status is positively associated with health-related quality of life [4–6]. Although work is beneficial to cancer survivors it also represents challenges. Cancer survivors might experience changes in their employment status [7], or work-related problems when they return to work, such as fatigue, cognitive limitations, impaired work ability, and changes in support [8–10]. Although some research has been done on which work-related problems are experienced by cancer survivors after their return to work [11], there is hardly any research on how those work-related problems might influence cancer survivors’ perceptions of their working life. Moreover, the objective outcomes of previous studies (e.g. work participation, productivity and work loss) [12–14] do not contribute to explaining this subjective outcome.

To evaluate the subjective working life of cancer survivors, we propose to study the quality of their working life, which is defined as ‘the experiences and perceptions of a cancer survivor in the work environment’. This definition is similar to other quality of working life definitions such as ‘capture the essence of an individual’s work experience in the broadest sense’ [15] and ‘corresponds to a condition experienced by the individual’ [16]. Previous research shows that a higher level of quality of working life in ‘healthy’ employees leads to lower levels of turnover intentions [17, 18]. Therefore, we presume that quality of working life is important to the process of return to work and work retention among cancer survivors.

To evaluate the quality of working life among working cancer survivors who have returned to work, we developed the Quality of Working Life Questionnaire for Cancer Survivors (QWLQ-CS) [19]. The QWLQ-CS is a subjective, self-administered questionnaire and contains items about general work issues, as well as cancer-specific items. The added value of this new questionnaire is that it measures more than just objective work outcomes, such as work limitations [20] or work ability in cancer survivors [21]. The QWLQ-CS measures how cancer survivors experience these work outcomes, and translates these subjective perspectives into a tangible concept: ‘Quality of Working Life’ (QWL). We developed this questionnaire because previous QWL questionnaires were developed for healthy employees [15, 16, 22, 23] or nurses [24]. These groups of employees differ in professional backgrounds, and they do not have the same medical background or accompanying work-related problems as cancer survivors who have returned to work. Consequently, we found the existing questionnaires to be less useful for this specific group of employees and we aimed to develop a new, valid and reliable QWL questionnaire.

We based the development of our QWL questionnaire on the guidelines for developing Questionnaire Modules set out by the European Organisation for Research and Treatment of Cancer (EORTC) Quality of Life Group [25]. The guidelines are widely used in the field of Quality of Life research [26], and provide researchers with a systematic approach to questionnaire development. The guidelines contain four phases in which development takes place. Our article described the pre-test phase of the initial QWLQ-CS.

The objective of this study was to pre-test the items in the QWLQ-CS for acceptability and comprehensibility. In addition, item retention was performed by pre-assessing the relevance scores and response distributions of the items in the QWLQ-CS. To use the QWLQ-CS as an evaluation instrument in further research or for interventions in occupational support, the questionnaire should be acceptable and comprehensible for multiple respondents from different ethnic, educational, and occupational backgrounds. Items should not be difficult, upsetting or ambiguous [25, 27]. Measuring the relevance of the items is one of the guidelines in developing questionnaires [25] because it is essential that the items represent issues that contribute to QWL of cancer survivors who have returned to work. Improving the relevance, acceptability and comprehensibility will contribute to good content validity of the QWLQ-CS [25, 28]. Pre-assessing the response distributions of the items was done because it would be preferable to avoid limiting the range of responses available to the cancer survivors who fill in the QWLQ-CS [29].

## Methods

### Study design

Cancer survivors who have returned to work filled in the initial QWLQ-CS, and they were later interviewed in a semi-structured manner. This study was based on phase 3 of the guidelines for developing Questionnaire Modules set out by the EORTC Quality of Life Group [25]. The aim of this phase was to pre-test the initial QWLQ-CS. For transparent reporting on the study and the semi-structured interviews, we used several items from the checklist of STrengthening the Reporting of OBservational studies in Epidemiology (STROBE statement) [30] (Additional file 1). Ethical approval for this study was requested but was not found necessary by the Medical Ethics Committee of the Academic Medical Center (W14_218#14.17.0264).

### Participants

Recruitment took place at an academic medical centre in the Netherlands from November 2014 to February 2015. Cancer survivors were approached if they: 1) had been diagnosed with lymphoma, leukaemia, prostate cancer, testicular cancer, breast cancer or gastrointestinal cancer; 2) were between 18 and 65 years of age; 3) were currently in paid employment or self-employed; 4) had been treated in the medical centre between 2004 and 2014; and 5) spoke and read Dutch fluently. An exclusion criterion was being diagnosed with cancer during childhood, as this group of cancer survivors might experience different (long-term) side effects from the cancer treatment and disadvantage in their general development (e.g. learning difficulties) [31]. Cancer survivors received an invitation letter by mail from their attending specialist, together with information about the study. If a cancer survivor was eligible and interested in participating, he or she could reply with a standardised form, which permitted a researcher from our department to contact the cancer survivor directly. The research assistant would then contact this person by phone and schedule an appointment at a preferred location, as indicated by the cancer survivor. The research assistant also approached a small group of cancer survivors (*N* = 15) who were originally recruited for our qualitative study [19], but who did not participate due to a variety of reasons, such as inability to attend the focus group meeting. As these cancer survivors remained interested in participating in research, they received information about the study by mail, with the request to indicate if they wished to participate in the current study. Subsequently, if a cancer survivor was eligible and willing to participate, a research assistant phoned or emailed this person and scheduled an appointment.

### Procedure

At various locations between December 2014 and March 2015, the cancer survivors filled in the initial QWLQ-CS. This was done in the presence of the first author (MdJ) or the research assistant. MdJ is a female PhD candidate working on the topic of cancer survivors, QWL and work continuation. The research assistant has a Master’s degree, and is involved in researching cancer survivors and their return-to-work or work continuation. The presence of the researchers enabled cancer survivors to ask any questions they might have while answering the items of the QWLQ-CS*.* Afterwards, MdJ or the research assistant interviewed the cancer survivors in a semi-structured manner by means of a topic list. This list contained such topics as acceptability and comprehensiveness of the items. The cancer survivors were encouraged to comment on items or provide additional concerns. All the cancer survivors signed an informed consent form, and gave their permission for the semi-structured interview to be audio-recorded. Prior to completing the initial QWLQ-CS, cancer survivors were asked to fill in a demographic questionnaire about their age, household composition, education, employment status, and their disease and treatment. After the appointment, we offered the cancer survivor a €15 voucher as a token of our appreciation.

#### The QWLQ-CS

The QWLQ-CS was originally developed in Dutch. The initial QWLQ-CS consisted of 102 items about general issues, work perceptions, job characteristics, social structure and environment, organisational characteristics, and effects of the disease and treatment [19, 32]. The list included both positively and negatively worded items, and response options were provided on a 4-point Likert scale without numbers in the following categories; 1) totally disagree; 2) disagree (a little); 3) agree (a little); and 4) totally agree. We provided the extra response option of ‘not applicable’ for cancer survivors who indicated that the item did not apply to their work or health situation. For instance, an item about supervisor support would not be applicable to a self-employed cancer survivor. Similarly, an item about difficult recovery in the previous four weeks would not be applicable to a cancer survivor who was diagnosed with cancer several years ago. To indicate if the items were relevant to their own QWL, cancer survivors rated each item for relevance on a 4-point Likert scale (1 = not relevant to 4 = very relevant).

#### Semi-structured interview

The acceptability of a questionnaire needs to be evaluated because the degree of acceptability determines if participants are willing to do something, such as answering all items [27]. Therefore, since cancer might be a confronting topic to some people, it is important to avoid confronting items that might upset people [25] and lead them to stop completing the QWLQ-CS. For this purpose, the interviewer started the semi-structured interview by asking the cancer survivor if any items were annoying or upsetting. If the cancer survivor reported an item, the interviewer asked for the reason and whether the cancer survivor would rather change or delete the item. Next, the interviewer took a closer look at the relevance of the items, and asked why some items were scored as ‘not relevant’. Finally, the interviewer and cancer survivor discussed the items that were difficult to answer, or confusing. As the target group of the QWLQ-CS is cancer survivors, it is important to gain information about items that improve the comprehensibility of the QWLQ-CS. To get a deeper understanding of why some items were difficult or confusing, the interviewer used verbal probe questions (e.g. detailed questions about perceived content and interpretation of the items) [27]. The interview was completed with debriefing questions about the clarity of the distribution, instruction and response scale, and if the cancer survivor believed relevant items were missing or redundant.

### Analyses

After the appointment, the researchers used the audio recording to complete their notes of the cancer survivors’ comments on the items from the initial QWLQ-CS. A data file was created that specified which items were annoying and upsetting, difficult, confusing, missing or redundant. The cancer survivors did not provide feedback on the findings. Since the research assistant would attend most appointments, MdJ checked 10 % of her notes, which were then compared with the audio recording. The response scores on the initial QWLQ-CS and the relevance ratings were analysed using IBM SPSS Statistics 20.

**Fig. 1:**
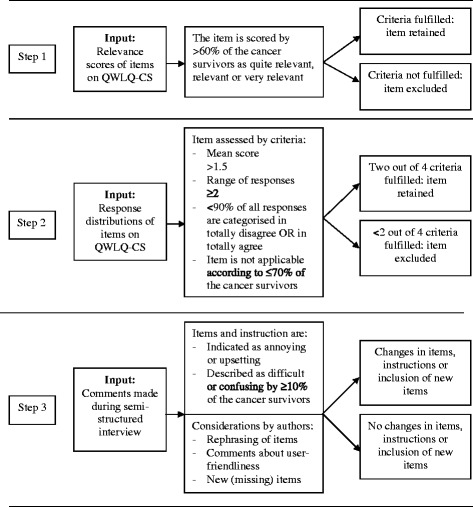
Decision rules for data analysis in three steps

Modified pre-set decision rules, based on the EORTC guidelines [25], were used to determine if items remained in the QWLQ-CS or should be changed by assessing relevance, acceptability, comprehensibility and item response distributions (Fig. 1). The first step was to analyse the relevance scores of the items provided by the cancer survivors. For an item to be regarded as relevant, the decision rule stated that >60 % of the cancer survivors should score an item as ‘quite a bit relevant’, ‘relevant’, or ‘very relevant’. In Step 2, responses on the items in the QWLQ-CS were analysed. Items were retained in the QWLQ-CS if they exceed at least two of the four requirements concerning the mean score, the response range, scores in the extreme lower and upper response categories, and scores in the ‘not applicable’ to work or health situation response category. The response distributions were assessed because it is not desirable to limit the range of responses available to the cancer survivors who fill in the QWLQ-CS [29]. Furthermore, assessing the response distributions of the items on the QWLQ-CS is also important for the psychometric properties of the questionnaire. For instance, if a high proportion of the total population has a score at the upper end of the scale, this item might affect the responsiveness of a questionnaire because they cannot score higher if any improvement occurs [27]. As stated, an item should meet at least two out of the four requirements in order to remain in the questionnaire. These requirements were; 1) mean score of >1.5; 2) response range of ≥2; 3) <90 % of all the responses fell in one of the categories 1 ‘totally disagree’ or 4 ‘totally agree’; and 4) item was indicated by ≤ 70 % of the cancer survivors as being ‘not applicable’. The items that were ‘not applicable’ to the cancer survivors were treated as missing variables in the analyses. The outcomes on the negative items in the QWLQ-CS were reversed as a result of the negative contribution to QWL. During the analyses, the reversed outcomes on those items were compared to the requirements. In Step 3, the acceptability and comprehensiveness of the items and the questionnaire instruction were assessed. Items needed to be changed if cancer survivors found them annoying or upsetting, and if ≥10 % of the cancer survivors stated that the item was difficult or confusing. The instruction of the QWLQ-CS was modified if 10 % or more of the cancer survivors made a remark about it being unclear. All other suggestions made by cancer survivors regarding rephrasing an item, the distribution of the QWLQ-CS or missing QWL items were considered by the researchers during a consensus meeting.

## Results

A total of 99 cancer survivors received an invitation to participate in the study based on the information available in the hospital (e.g. age, diagnosis and time of treatment). Not all of these cancer survivors were in paid employment or self-employment, could speak and read Dutch fluently, or were interested in participation, so a smaller number of cancer survivors fulfilled the inclusion criteria or responded. In the end, 21 cancer survivors participated in the interviews. Data on two cancer survivors were excluded from analyses due to current sick leave and failure to accurate representing the work situation within the last four weeks (*N* = 1), and due to answers heavily influenced and corrected by the spouse who was present during the interview (*N* = 1). During all other appointments, only the researcher and the cancer survivor were present (*N* = 19). The appointments with the cancer survivors took place in their homes (*N* = 9), at the hospital (*N* = 9), or at their workplace (*N* = 1). The duration of the appointments ranged from 40–90 min.

### Cancer survivors’ characteristics

Of the 19 cancer survivors, 53 % were male (*N* = 10); the mean age was 51 ± 11 years old (range 25–62 years). Table 1 displays the diagnoses of the cancer survivors: lymphoma (16 %), leukaemia (21 %), prostate cancer (16 %), breast cancer (26 %), and colon cancer (26 %). The majority of the cancer survivors were diagnosed with cancer between 2009 and 2013, and received treatments in surgery (58 %), chemotherapy (63 %) and radiotherapy (68 %). Most worked full-time (58 %), and had a permanent employment contract (79 %) within a variety of occupational sectors, including banking (16 %), and corporate services (16 %). The participants’ specific jobs were: attorney/mediator, business manager in syndications, credit services employee, director of management development, home carer, insurance adviser, investment analyst, IT consultant, management assistant, management consultant, nurse/care coordinator, police officer, product manager, programme manager, quality adviser, swimming instructor/supervisor, test engineer, treasurer and self-employed food retail professional.

**Table 1:** Characteristics of the cancer survivors

		*N* = 19	
Demographic characteristics			
Age (mean in years ± SD)		51 ± 11	
		N	(%)
Gender - male		10	(53)
Marital status	Married/living with a partner	17	(90)
	Single	2	(11)
Country of origin	Holland	15	(79)
	India	1	(5)
	Singapore	1	(5)
	Ghana	1	(5)
	Surinam	1	(5)
Clinical characteristics			
Diagnosis	Lymphoma	3	(16)
	Leukaemia	4	(21)
	Prostate cancer	3	(16)
	Breast cancer	5	(26)
	Colon cancer	5	(26)
	Laryngeal cancer	1	(5)
Year of cancer diagnosis	1995	1	(5)
	2007	1	(5)
	2009	3	(16)
	2010	3	(16)
	2011	5	(26)
	2012	6	(32)
	2013	2	(11)
Treatment	Surgery	11	(58)
	Chemotherapy	12	(63)
	Radiotherapy	13	(68)
	Other (e.g. stem cell transplant, hormone therapy)	6	(32)
Work characteristics			
Education	Secondary education	4	(21)
	Intermediate vocational education	3	(16)
	Higher professional education	6	(32)
	Academic education	6	(32)
Work contract	Permanent position	15	(79)
	Self-employed	4	(21)
Contract hours	Full-time	11	(58)
	Part-time (12–32 hours)	7	(37)
Breadwinner position	Sole or shared	17	(89)
Occupational sector	Banking	3	(16)
	Health care and pharmacy	1	(5)
	Public services	1	(5)
	Facility management	1	(5)
	Trade and retail businesses	1	(5)
	ICT	2	(11)
	Legal/juridical	1	(5)
	Police/security	1	(5)
	Educational	1	(5)
	Corporate services	3	(16)
	Other	4	(21)

### Outcomes

#### Step 1: Relevance scores

The percentages of cancer survivors who scored the items as relevant ranged from 65 to 100 %. The item that was scored relevant by the lowest percentage of cancer survivors was ‘my colleagues feel pity for me’ (65 %). Of the 102 items, all cancer survivors scored a total of 56 items as relevant. Everyone found the item ‘I really want to work’ relevant. The remaining 46 items were found relevant by 65 to 95 % of the cancer survivors, such as the item ‘I think it is necessary to work’. The outcome of Step 1 was that all items were scored as relevant by more than 60 % of the cancer survivors, so all items remained in the QWLQ-CS after Step 1.

#### Step 2: Response distributions

The original mean scores of the items in the QWLQ-CS varied from 1.6 to 4.0. In Table 2, we provide a summary of the mean scores per category. It displays more items than were included in the initial QWLQ-CS, but this outcome is elaborated in Step 3. The items that had the lowest mean scores stated ‘My colleagues have high expectations of me’ or ‘My supervisor has high expectations of me’ (mean: 1.6). Originally, these items both scored a round mean score of 1.6 in the QWLQ-CS, but due to the negative contribution to QWL, the mean scores of these items were reversed to 3.5 and 3.6. The positively phrased item with the highest mean score of 4.0 was ‘I appreciate my work’. All items fulfilled the criteria of obtaining a mean score of 1.5.

**Table 2:** Summary of the 104-item preliminary version of the QWLQ-CS

Categories	Number of items	Mean of items	Phrase item	Examples
General feelings about working life	12	2.8 – 4.0	+^1^	Satisfaction
				Enjoying work
				Appreciation of work
				Importance of work
				Meaning of work
Job characteristics	18	3.1 – 4.3	+	Good work circumstances
				Satisfaction with work hours and (temporary) adjustments
				Accessible workplace
				Satisfaction with current income
				Career opportunities
	1	2.0	−^2^	Travel time from home to work
Social structure and culture	11	3.1 – 4.0	+	Good contacts with people in work environment
				Feelings of being valued
				Involvement with the organisation
				Safe and/or trusting atmosphere
				Disclosure
Contact with supervisor	5	4.0 – 4.1	+	Relationship with supervisor
				Understanding and support from supervisor
				Communication with supervisor
	1	3.6	−	Supervisor’s expectations
Contact with colleagues	5	3.7 – 4.0	+	Relationships with colleagues
				Understanding and support from colleagues
				Communication with colleagues
	4	1.4 – 3.5	−	Colleagues’ expectations
				Taboos in the workplace
Contact with other actors at work	9	3.5 – 4.2	+	Support from specialist
				Support from occupational physician
				Contact with customers/clients
				Communication with Human Resources department
	2	1.7	−	Occupational physician’s attitude
Organizational characteristics	5	3.6 – 4.2	+	Organisational involvement
				Communication about work adjustments
				Company regulations
	1	2.5	−	Openness within organisation about general course of events
Work perceptions	8	3.5 – 4.0	+	Productivity
				Self-confidence in work
				Estimation of performance
				In charge of own duties
	7	1.1 – 2.8	−	Vulnerability in work
				High workload
				Work stress
Effects of the disease and treatment	2	3.5 – 4.1	+	Acceptation of health situation and possible limitations
	8	2.0 – 2.8	−	Work limitations
				Uncertainty about the future
				Difficult recovery
				Concentration and/or memory problems in work
				Fatigue and/or shortage of energy in work
				Fear of change in job function
Private life	3	3.1 – 3.7	+	Work-life balance
				Work support in private life
	2	1.9 – 3.1	−	Expectations of private life

^1^ Positively phrased items, ^2^ Negatively phrased items

A total of 40 items (39 %) failed to meet the criteria of covering a range of two or more points. None of the items had a four-point range; the majority had a range of two (26 items) or three (36 items). An item with a range of two was ‘I am pleased to determine my own work hours’, while ‘My career opportunities are good’ had a three-point range. The items that had a range of one (39 items) discussed general feelings about the work life and social support from colleagues and supervisors. Examples include ‘I enjoy my work’ and ‘I receive support from my supervisor’.

The analysis of the upper and lower scores of the items resulted in five items not meeting the criteria of scoring <90 % of all the responses in the response category 1 ‘totally disagree’ or response category 4 ‘totally agree’. The item that scored 95 % in the first category ‘totally disagree’ was ‘I feel helpless’. The other four items that scored <90 % in the fourth category ‘totally agree’ were; 1) ‘I am satisfied with my work’; 2) ‘I am suitable for my work’; 3) I am performing; and 4) ‘I appreciate my work’. In that last group, all cancer survivors (100 %) agreed fully.

Some items were scored as being not applicable by a percentage of cancer survivors. These items contained statements about adjustments in work, colleagues and supervisors. Nevertheless, no more than 70 % of the cancer survivors scored any item on the QWLQ-CS as ‘not applicable’. Combining the outcomes on the above criteria, no items were excluded from the QWLQ-CS because they met at least two out of four criteria in Step 2.

#### Step 3: Comments in semi-structured interviews

All cancer survivors’ comments were analysed, as no items were excluded in the previous steps. In Step 3, none of the items were indicated as annoying or upsetting. In the case of 73 items, more than 10 % of cancer survivors (*N* = 2) stated that the item was difficult or confusing. We added the words ‘in the work situation/at work’ to clarify 13 items, such as ‘I feel vulnerable at work’. Also items were merged (6 items) if they were considered twofold, while others were separated (5 items) if they were considered confusing. For instance, the items ‘I feel involved with the organisation’ and ‘I feel connected to the organisation’ were combined, and reformulated as ‘I feel involved, as well as connected to the organisation’ (Table 2). One item that was separated was ‘I receive support from my specialist and/or occupational physician’. In the revised QWLQ-CS, the two items refer to either the specialist or the occupational physician. Furthermore, we changed the word ‘job’ into ‘function’ in the item ‘I am confident that I can continue to work in this function’. Some of the cancer survivors commented on the item ‘I am satisfied with the adjustments in my work duties’. Since these adjustments are not always definite we added ‘(temporary)’ in front of this word. In accordance with cancer survivors’ comments, we clarified the introduction and emphasised that the items must be answered about the work situation in the previous four weeks. We also explained more explicitly when to use the response category ‘not applicable’ by giving separate example for cancer survivors who were employees and for cancer survivors who were self-employed. For instance, in the case of a self-employed cancer survivor, the items that refer to a supervisor do not apply to them or their specific situation. Some comments for rephrasing words were incorporated, such as the term ‘health situation’ instead of ‘disease’. We also adjusted the order of the QWLQ-CS to make the questionnaire more user-friendly. Finally, the authors discussed the missing issues that were mentioned, but none were added to the existing list of items. One of the missing issues was about the process of treatment in the hospital, and how a negative experience (e.g. mistakes during treatment) might lead to more physical problems and lower quality of working life. These physical problems and their consequences are also addressed by issues in the category ‘effects of the disease and treatment’, such as ‘difficult recovery’, ‘work limitations’ and ‘fatigue and/or shortage of energy in work’. Therefore, we presume that the items about the effects of the disease and treatment are sufficient to address this issue.

## Discussion

The objective of this study was to pre-test the items of the initial Quality of Working Life Questionnaire for Cancer Survivors (QWLQ-CS) for acceptability and comprehensiveness. In addition, item retention was performed by pre-assessing the relevance scores and response distributions of the items on the QWLQ-CS. Item retention was based on pre-set decision rules. All items met the decision rules for relevance and response distributions, and were therefore retained in the QWLQ-CS. To improve acceptability and comprehensiveness, some items were adjusted and rephrased. This resulted in a 104-item preliminary version of the QWLQ-CS.

### QWLQ-CS

The items on the QWLQ-CS were scored as relevant by 65–100 % of the cancer survivors, and this high percentage was not unexpected. It is most likely due to the guidelines on questionnaire development we followed, and its emphasis on the importance of item relevance. In this study, the items with the lowest scores on relevance (<80 %) were the negatively phrased items; ‘I feel helpless’, ‘My colleagues feel sorry for me’, ‘My occupational physician and/or the department Human Resources department have a negative attitude towards me’, ‘I experience taboos in the workplace’, I have negative encounters with my colleagues’. One possible explanation for these lower relevance scores is that this sample of cancer survivors had a reasonably ‘high’ QWL. Cancer survivors who are happy at work, are satisfied and do not experience work problems might find negative items that imply difficulties at the workplace less relevant. The items that were found relevant by the entire sample (100 %) were items about general feelings of the working life such as ‘I think my work is important’, ‘I have positive feelings about my work’, or ‘I appreciate my work’. Furthermore, the cancer survivors also found items about their work perceptions relevant. For instance, ‘I have the feeling I can be myself’, ‘I feel connected to the organisation’, or ‘Work gives structure to my life’. Compared to the item that was found relevant by somewhat fewer cancer survivors, these items are very general.

The QWLQ-CS was developed as a generic questionnaire for cancer survivors. As some items in this study were indicated by cancer survivors as being ‘not applicable’ to their work or health situation, it could be argued that the QWLQ-CS is too generic and should be split accordingly. This is possible if the work situation was the reason for choosing the ‘not applicable’ response option, because the items in the QWLQ-CS can be split into one part for cancer survivors who work as employees within an organisation and another part for cancer survivors who are self-employed. This is less simple for the items regarding health situations, however, since not all cancer survivors experience limitations to the same extent. Instead, one could consider specific cancer diagnoses and their known corresponding limitations in adjusting the QWLQ-CS. For instance, the disease-specific health-related quality of life questionnaire (THYCA-QoL) for thyroid cancer [33] is a supplement to the more general EORTC Quality of Life Questionnaire (EORTC QLQ-C30) [34]. Nevertheless, these considerations should be postponed to a later stage, after the psychometric properties have been tested and the number of items have been reduced.

### Practical relevance

This pre-tested version of the QWLQ-CS might have practical relevance to occupational physicians, employers or patients who acknowledge the importance of psychological well-being and job satisfaction in workers [35]. Although, the preliminary QWLQ-CS cannot be administered to cancer survivors who have returned to work yet, this version of the QWLQ-CS does provide important issues that contribute to the QWL of cancer survivors. These QWL issues can already serve as guidelines for occupational physicians or employers who support cancer survivors in returning to work or continuing their work. The QWLQ-CS might offer them QWL topics that they can discuss with cancer survivors in order to identify any improvements in their work situation.

### Implications for research

This pre-test study was the third phase in the development of the QWLQ-CS, in which the relevance, acceptability and comprehensibility of the items were tested. Next, in the fourth and final phase, a field study will be conducted to test if the QWLQ-CS is a reliable, valid and responsive instrument to measure QWL among cancer survivors. After this final phase, in which the psychometric properties of the fully developed QWLQ-CS will be tested, it can be implemented in research for evaluative purposes. For instance, it could be used in the evaluation of a new or current intervention that aims at improving the QWL of cancer survivors who have returned to work. By then, we may also indicate if the QWLQ-CS can be used as a tool to distinguish between cancer survivors with low and high quality of working life. Further research might be conducted by allocating specific interventions for work support to specific groups of cancer survivors.

### Strengths and limitations

The main strength of this study is that we systematically assessed the relevance, and improved the acceptability and comprehensiveness of the initial QWLQ-CS. We based our study design on guidelines for questionnaire development that are widely used by other researchers in the field of oncology [36]. First and foremost, we accurately assessed relevance in the development of the QWLQ-CS. In our previous qualitative study, in which we retrieved and selected relevant QWL issues for the initial QWLQ-CS [19], we included a different sample of cancer survivors than in this pre-test study. In both studies, we asked the cancer survivors to score the items on relevance. The outcome in this pre-test study contributes to the assumption that the items in the initial QWLQ-CS are indeed relevant to cancer survivors who have returned to work; more than 60 % of the cancer survivors scored the items as relevant. In improving the acceptability and comprehensiveness of the QWLQ-CS, using a new sample in this pre-test study contributed to identifying potential new problems, as the cancer survivors were being introduced to the topic of QWL and were not already familiar with it. The design of this study provided the cancer survivors with the opportunity to discuss potential problems. While cancer survivors were filling in the QWLQ-CS, they were able to think out loud and discuss unclear items with the researcher. The ‘thinking aloud’ method is well known and is used to explore possible problems in depth [37]. This contributed to improving the acceptability of the questionnaire. Acceptability is important, since it increases the willingness of patients to participate. If the questionnaire is acceptable to cancer survivors, this also maximises response rates [38].

Another strength of the study was the diversity of the sample of cancer survivors, since the diagnoses of cancer varied, as did the time since diagnosis. Nevertheless, while analysing the sample, we also noticed similarities in the education level of the cancer survivors and in which occupational sector they worked. More cancer survivors received a higher education level, and performed more white-collar jobs. This similarity will not influence the content of the questionnaire since we did not exclude any items. However, it might have influenced the wording of the items, as we modified and rephrased them based on the comments of the cancer survivors. A rule of thumb is that the items should not require reading skills beyond that of a 12-year-old [39]. Due to the observation that more highly educated cancer survivors participated, the phrasing of the items might be too complicated for less well educated workers with cancer. On the other hand, we were aware of this possible threat to the generalisability of the QWLQ-CS, so we mutually discussed the phrasing of the items carefully.

A limitation was that the questionnaire was quite challenging for some cancer survivors. First, they were asked to indicate on a 4-point Likert scale how much they agreed or disagreed with an item about their work situation in the previous four weeks. Second, they immediately needed to score the same item on relevance for their QWL in general. The translation from, for instance, do I ‘totally disagree’ to ‘totally agree’ with this item in the previous four weeks, to is it ‘not relevant’ to ‘very relevant’ for my quality of working life in general, was confusing. For instance, it might happen that a cancer survivor agreed with the item because it happened at work in the previous four weeks, but did not consider it relevant to his or her QWL in general. The possibility of agreeing with the item but not finding it relevant sometimes caused confusion. A possible explanation for this confusion is that people try to appear consistent. If their answer to an item seems to contradict the previous answer, they will want to return to that previous item and alter it in order to restore the consistency [39]. As quite a few cancer survivors asked for confirmation and modified their answers after clarification, the results in this study might be influenced by the design of the questionnaire. But then, as we did not exclude issues but only modified them, we assume that this does not have consequences for the preliminary version of the QWLQ-CS.

## Conclusions

The Quality of Working Life Questionnaire for Cancer Survivors (QWLQ-CS) consists of 104 items about the experiences and perceptions of a cancer survivor in the work environment. The items are relevant, acceptable and comprehensible for cancer survivors. There was no evident indication for uneven response distributions of the items. The preliminary QWLQ-CS is now suitable for large sample sizes of cancer survivors, which is necessary to test the psychometric properties of the questionnaire and to develop the final QWLQ-CS.

## Abbreviations

EORTC, European Organisation for Research and Treatment of Cancer; QWL, Quality of working life; QWLQ-CS, Quality of Working Life Questionnaire for Cancer Survivors; STROBE statement, STrengthening the Reporting of OBservational studies in Epidemiology.

## Additional file

Additional file 1: STROBE Statement—checklist of items that should be included in reports of observational studies. (DOC 89 kb)

## References

[1] Joutard X, Paraponaris A, Sagaon Teyssier L, Ventelou B (2012). Continuous-time Markov model for transitions between employment and non-employment: the impact of a cancer diagnosis. Ann Econ Stat.

[2] Rowland JH, Bellizzi KM (2014). Cancer survivorship issues: life after treatment and implications for an aging population. J Clin Oncol.

[3] Bouhnik AD, Bendiane MK, Cortaredona S, Sagaon Teyssier L, Rey D, Berenger C (2015). The labour market, psychosocial outcomes and health conditions in cancer survivors: protocol for a nationwide longitudinal survey 2 and 5 years after cancer diagnosis (the VICAN survey). BMJ Open.

[4] Lilliehorn S, Hamberg K, Kero A, Salander P (2013). Meaning of work and the returning process after breast cancer: a longitudinal study of 56 women. Scand J Caring Sci.

[5] Johnsson A, Fornander T, Rutqvist LE, Olsson M (2010). Factors influencing return to work: a narrative study of women treated for breast cancer. Eur J Cancer Care (Engl).

[6] Bieri S, Roosnek E, Helg C, Verholen F, Robert D, Chapuis B (2008). Quality of life and social integration after allogeneic hematopoietic SCT. Bone Marrow Transplant.

[7] Tevaarwerk AJ, Lee JW, Sesto ME, Buhr KA, Cleeland CS, Manola J (2013). Employment outcomes among survivors of common cancers: the Symptom Outcomes and Practice Patterns (SOAPP) study. J Cancer Surviv.

[8] Duijts SF, van Egmond MP, Spelten E, van Muijen P, Anema JR, van der Beek AJ (2014). Physical and psychosocial problems in cancer survivors beyond return to work: a systematic review. Psychooncology.

[9] Banning M (2011). Employment and breast cancer: a meta-ethnography. Eur J Cancer Care (Engl).

[10] Braybrooke JP, Mimoun S, Zarca D, Elia D, Pinder B, Lloyd AJ (2015). Patients’ experiences following breast cancer treatment: an exploratory survey of personal and work experiences of breast cancer patients from three European countries. Eur J Cancer Care (Engl).

[11] Tamminga SJ, de Boer AG, Verbeek JH, Frings-Dresen MH (2012). Breast cancer survivors’ views of factors that influence the return-to-work process- a qualitative study. Scand J Work Environ Health.

[12] Blinder V, Patil S, Eberle C, Griggs J, Maly RC (2013). Early predictors of not returning to work in low-income breast cancer survivors: a 5-year longitudinal study. Breast Cancer Res Treat.

[13] Nugent BD, Weimer J, Choi CJ, Bradley CJ, Bender CM, Ryan CM (2014). Work productivity and neuropsychological function in persons with skull base tumors. Neuro Oncol Pract.

[14] Pearce A, Timmons A, O’Sullivan E, Gallagher P, Gooberman-Hill R, Thomas AA (2015). Long-term workforce participation patterns following head and neck cancer. J Cancer Surviv.

[15] van Laar D, Edwards JA, Easton S (2007). The work-related quality of life scale for healthcare workers. J Adv Nurs.

[16] Martel J-P, Dupuis G (2006). Quality of Work Life: Theoretical and Methodological Problems, and Presentation of a New Model and Measuring Instrument. Soc Indic Res.

[17] Chan KW, Wyatt TA (2007). Quality of work life: A study of employees in Shanghai, China. Asia Pacific Bus Rev.

[18] Mosadeghrad AM (2013). Quality of working life: an antecedent to employee turnover intention. Int J Health Policy Manag.

[19] de Jong M, Tamminga SJ, de Boer AG, Frings-Dresen MH (2016). Quality of working life of cancer survivors: development of a cancer-specific questionnaire. J Cancer Surviv.

[20] Tamminga SJ, Verbeek JH, Frings-Dresen MH, de Boer AG (2014). Measurement properties of the Work Limitations Questionnaire were sufficient among cancer survivors. Qual Life Res.

[21] de Boer AG, Verbeek JH, Spelten ER, Uitterhoeve AL, Ansink AC, de Reijke TM (2008). Work ability and return-to-work in cancer patients. Br J Cancer.

[22] Ventegodt S, Andersen NJ, Kandel I, Enevoldsen L, Merrick J (2008). Scientific research in the quality of working-life (QWL): generic measuring of the global working life quality with the SEQWL questionnaire. Int J Disabil Hum Dev.

[23] Murphy L, Perrewé PL, Ganster DC (2002). Job stress research at NIOSH: 1972–2002. Research in occupational stress and well-being: historical and current perspectives on stress and health. 2.

[24] Brooks BA, Anderson MA (2005). Defining quality of nursing work life. Nurs Econ.

[25] Johnson C, Aaronson N, Blazeby JM, Bottomley A, Fayers P, Koller M, et al. EORTC quality of life group: guidelines for developing questionnaires modules Brussels: EORTC; 2011 [cited 2015 8 August]. Available from: http://groups.eortc.be/qol/sites/default/files/archives/guidelines_for_developing_questionnaire-_final.pdf.

[26] Holzner B, Efficace F, Basso U, Johnson CD, Aaronson NK, Arraras JI (2013). Cross-cultural development of an EORTC questionnaire to assess health-related quality of life in patients with testicular cancer: the EORTC QLQ-TC26. Qual Life Res.

[27] de Vet HC, Terwee CB, Mokkink LB, Knol DL (2011). Measurement in medicine. A practical guide.

[28] Brod M, Tesler LE, Christensen TL (2009). Qualitative research and content validity: developing best practices based on science and experience. Qual Life Res.

[29] Land L, Ross JD (2014). Barriers to questionnaire completion: understanding the AIDS/HIV patient’s perspective. Nurse Res.

[30] von Elm E, Altman DG, Egger M, Pocock SJ, Gotzsche PC, Vandenbroucke JP (2014). The Strengthening the Reporting of Observational Studies in Epidemiology (STROBE) Statement: guidelines for reporting observational studies. Int J Surg.

[31] Howard A, Goddard K, Tan de Bibiana J, Pritchard S, Olson R, Kazanjian A. Adult childhood cancer survivors’ narratives of managing their health: the unexpected and the unresolved. J Cancer Surviv. 2016; doi:10.1007/s11764-016-0517-8. 10.1007/s11764-016-0517-8 PMC4920835 26833205

[32] de Jong M, de Boer AG, Tamminga SJ, Frings-Dresen MH (2015). Quality of working life issues of employees with a chronic physical disease: a systematic review. J Occup Rehabil.

[33] Husson O, Haak HR, Mols F, Nieuwenhuijzen GA, Nieuwlaat WA, Reemst PH (2013). Development of a disease-specific health-related quality of life questionnaire (THYCA-QoL) for thyroid cancer survivors. Acta Oncol.

[34] Aaronson NK, Ahmedzai S, Bergman B, Bullinger M, Cull A, Duez NJ (1993). The European Organization for Research and Treatment of Cancer QLQ-C30: a quality-of-life instrument for use in international clinical trials in oncology. J Natl Cancer Inst.

[35] Wright TA, Bonett DG (2007). Job satisfaction and psychological well-being as nonadditive predictors of workplace turnover. J Manage.

[36] Winters ZE, Balta V, Thomson HJ, Brandberg Y, Oberguggenberger A, Sinove Y (2014). Phase III development of the European Organization for Research and Treatment of Cancer Quality of Life Questionnaire module for women undergoing breast reconstruction. Br J Surg.

[37] Ericsson KA, Simon HA (1980). Verbal reports as data. Psychol Rev.

[38] Williams A (2003). How to…write and analyse a questionnaire. J Orthod.

[39] Streiner DL, Norman GR (2008). Health measurement scales: a pratical guide to their development and use.

